# Enhanced recovery programmes in hepatobiliary and pancreatic surgery: a systematic review

**DOI:** 10.1308/003588412X13171221592410

**Published:** 2012-07

**Authors:** TC Hall, AR Dennison, DK Bilku, MS Metcalfe, G Garcea

**Affiliations:** University Hospitals of Leicester NHS Trust,UK

**Keywords:** Systematic review, Enhanced recovery, Fast track surgery, Hepatobiliary surgery, Pancreatic surgery, Liver surgery

## Abstract

**INTRODUCTION:**

The terms ‘enhanced recovery after surgery’, ‘enhanced recovery programme’ (ERP) and ‘fast track surgery’ refer to multimodal strategies aiming to streamline peri-operative care pathways, to maximise effectiveness and minimise costs. While the results of ERP in colorectal surgery are well reported, there have been no reviews examining if these concepts could be applied safely to hepatopancreatobiliary (HPB) surgery. The aim of this systematic review was to appraise the current evidence for ERP in HPB surgery.

**METHODS:**

A MEDLINE® literature search was undertaken using the keywords ‘enhanced recovery’, ‘fast-track’, ‘peri-operative’, ‘surgery’, ‘pancreas’ and ‘liver’ and their derivatives such as ‘pancreatic’ or ‘hepatic’. The primary endpoint was length of post-operative hospital stay. Secondary endpoints were morbidity, mortality and readmission rate.

**RESULTS:**

Ten articles were retrieved describing an ERP. ERP protocols varied slightly between studies. A reduction in length of stay was a consistent finding following the incorporation of ERP when compared with historical controls. This was not at the expense of increased rates of readmission, morbidity or mortality in any study.

**CONCLUSIONS:**

The introduction of an ERP in HPB surgery appears safe and feasible. Currently, many of the principles of the multimodal pathway are derived from the colorectal ERP and distinct differences exist, which may impede its implementation in HPB surgery.

‘Enhanced recovery after surgery’ or ‘fast track surgery’ pathways aim to streamline peri-operative care delivery and maximise effectiveness while minimising costs. They represent multimodal strategies that include patient education, optimal analgesic relief, stress reduction with regional anaesthesia, focused nursing and early mobilisation to augment the rapid return of functional recovery.^1,2^ They also represent a paradigm shift from traditional surgical philosophies and incorporate the use of minimally invasive methods and fewer or no surgical drains.

Enhanced recovery programmes (ERPs) have been the subject of numerous systematic reviews in colorectal surgery and most have demonstrated reduced post-operative stay, lower complication rates and reduced hospital costs, leading to their increasing use.^2–5^ There are also reports demonstrating improved outcomes with the use of similar pathways in vascular^6,7^ and urological^8,9^ procedures. However, peri-operative strategies with a strong evidence base supporting its use are not yet implemented widely in hepatopancreatobiliary (HPB) surgery.

Post-operative stay after pancreatic or liver resection is usually 12–17 and 8–14 days respectively at high volume centres.^10–14^ Pancreatic resection has always been considered a high risk procedure with an associated morbidity and mortality of 30–60% and 5% respectively.^10,11^ Liver resection too is considered high risk, and has an associated morbidity and mortality of 38–45% and 2.7–3.1% respectively.^13,14^

Controversy exists over the role of an ERP in HPB surgery. There have been no previous systematic reviews conclusively proving whether such concepts could be applied safely to such complex and major abdominal surgery. The aim of this systematic review was to appraise the current evidence for the incorporation of an ERP for major pancreatic and hepatic resections.

## Methods

A MEDLINE® literature search was undertaken using the keywords ‘enhanced recovery’, ‘fast-track’, ‘peri-operative’, ‘surgery’, ‘pancreas’ and ‘liver’ and their derivatives such as ‘pancreatic’ or ‘hepatic’. The inclusion criteria were studies examining the impact of fast track surgery on outcomes in any HPB surgery. Studies were included if they incorporated a sufficient description of the multimodal clinical ERP together with the required outcome measures. Studies were excluded if they examined only a single intervention in peri-operative management outside the context of an ERP. The search was limited to English language manuscripts only. All articles retrieved had the references cross-checked to ensure capture of cited pertinent articles. The primary endpoint was length of post-operative hospital stay. Secondary endpoints were morbidity, mortality and readmission rate. The evidence that established each element of the pathway was not the purpose of this review and is not discussed further.

## Results

**Figure 1:**
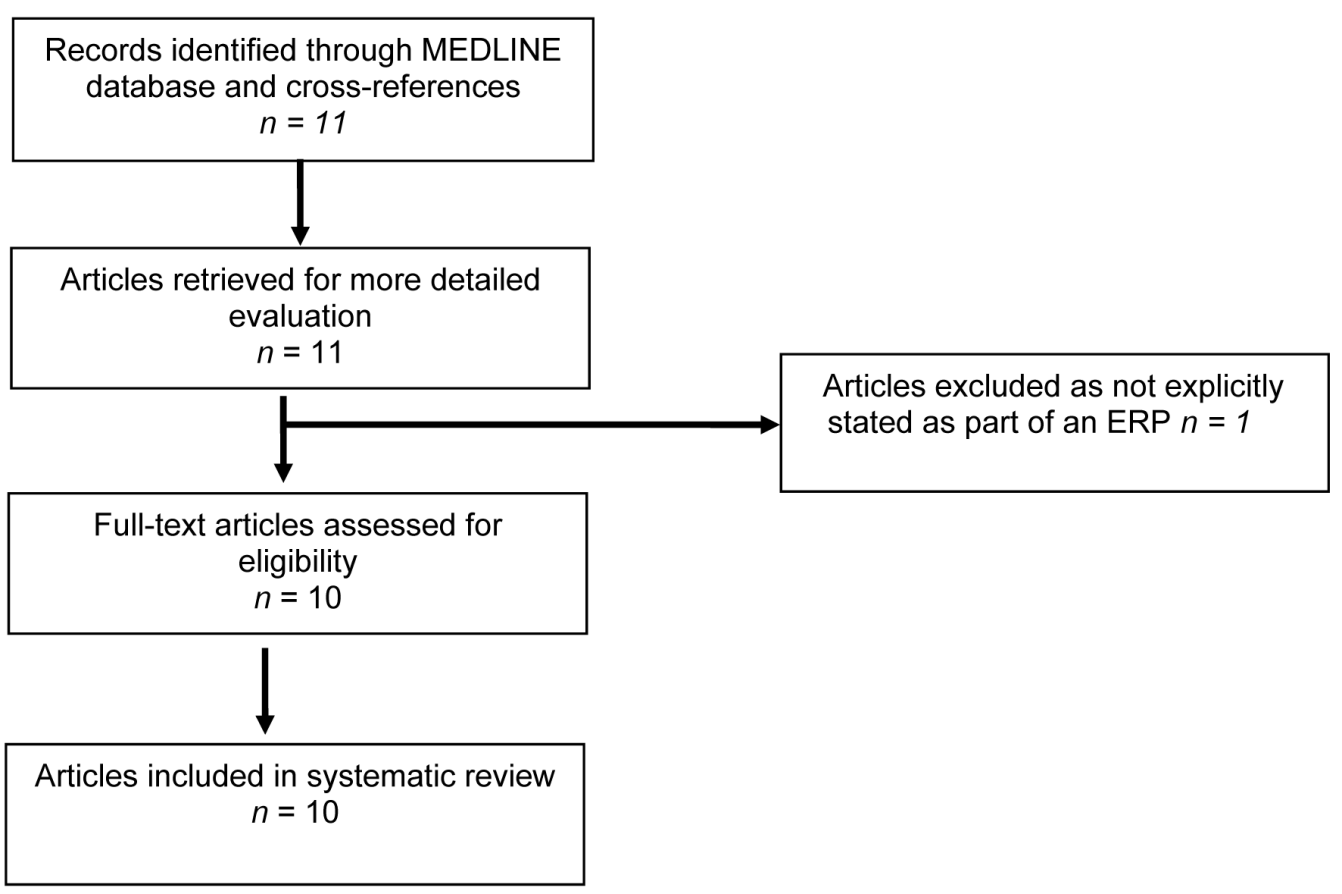
Flow diagram for the systematic review

A total of 11 articles, published between 2007–2011, were retrieved that met the inclusion criteria.^15–25^ One article that described a randomised controlled trial (RCT) of early enteral nutrition in patients undergoing major upper gastrointestinal surgical resection was excluded as the patients were not explicitly described as being part of an ERP (Fig 1).^15^ Table 1 shows a summary of the remaining ten articles. Two articles describing a single intervention in one parameter of peri-operative care but within an ERP were included.^21,22^ One of these studies comprised an RCT of laxatives and oral nutritional supplements following liver resection.^22^ The other investigated the effects of analgesia with single dose intrathecal morphine with gabapentin or continuous epidural analgesia.^21^

**Table 1:** Articles describing the enhanced recovery programme in hepatobiliary and pancreatic surgery

Authors	Year	Surgery (liver / pancreas)	Study design	Surgery type (ERP cohort where applicable)	Patients in ERP	Significant study findings compared with historical control
Berberat *et al*^16^	2007	Pancreas	Prospective historical comparison study	Pancreatic head resection 70.6%; distal 20%; total 5.9%; segmental 3.5%	255	
Balzano *et al*^17^	2008	Pancreas	Prospective historical comparison study	PD	252	More rapid time to passing first stool (5 vs 6 days, *p*<0.001); shorter length of stay (13 vs 15 days, *p*<0.001); less morbidity (47.2% vs 58.7%, *p*=0.014) with no difference in readmission rate (7.1% vs 6.3%, *p*=0.865)
van Dam *et al*^18^	2008	Liver	Prospective case series comparing with a historical control	Hemihepatectomy 33%; hemihepatectomy + metastasectomy 10%; extended hemihepatectomy 11%; multisegmental 28%; central resection 2%; metastasectomy 16%; repeat hepatectomy 11%	61	Reduced length of stay (6 vs 8 days, *p*<0.001); no significant difference in morbidity (41% vs 31%, *p*=0.197) or readmission rate (13% vs 10%, *p*=0.61)
MacKay *et al*^19^	2008	Liver	Prospective case series	1 lobectomy; 2 trisegmentectomy; 3 bisegmentectomy; 6 segment	12	
Stoot *et al*^20^	2009	Liver	Prospective multlcentre comparison study	Laparoscopic lateral resection, 1 segment IV	13	No significant reductions in length of stay (5 vs 7 days, *p*=0.305) or morbidity/mortality; significantly less intra-operative blood loss (50ml vs 250ml, *p*=0.002)
Koea *et al*^21^	2009	Liver	Consecutive patients in an ERP comparing analgesia with single dose intrathecal morphine with gabapentin or continuous epidural analgesia	Hemihepatectomy 36%; extended hepatectomy 4%; multisegementectomy 18%; monosegmentectomy 5%; metastasectomy 22%	50	
Hendry *et al*^22^	2010	Liver	Randomised controlled trial of laxatives and oral nutrition supplements within an ERP	Major resection 77.9%; minor resection 22.1%	68	
Montiel Casado *et al*^23^	2010	Pancreas	Retrospective historical comparison study	Classic PD	82	
di Sebastiano *et al*^24^	2011	Pancreas	Prospective historical comparison study	Pylorus preserving PD 62.1%; PD 2.7%; duodenum preserving pancreatic head resection 2.7%; distal pancreatectomy 13.8%; central pancreatectomy 2.1%; total pancreatectomy 6.9%; completion pancreatectomy 1.4%; other 8.3%	145	
Lin *et al*^25^	2011	Liver	Prospective comparison study at same site before and after introduction of ERP	Blsegmentectomy 30.4%; segmentectomy 23.2%; hemihepatectomy 16.1%; non- anatomlcal resection 12.5%; central resection 10.7%; extended hemlhepatectomy 7.1%	61	Reduced length of stay (7 vs 11 days, *p*<0.01); no difference In morbidity (37.7% vs 37.5%, *p*=0.982), mortality (1.8% vs 1.6%, *p*=0.706) or readmission rate (7.1% vs 3.3%, *p*=0.424)

ERP = enhanced recovery programme; PD = pancreaticoduodenectomy

Fast track surgery was described in six articles in liver resections^18–22,25^ and in four articles in pancreatic surgery.^16,17,23,24^ A total of 734 patients were included having had pancreatic surgery and a total of 265 patients after liver resection.

Six studies were prospective case series that compared outcomes of the ERP with historical controls, not necessarily in the authors’ institution.^16–19,24,25^ One study was a retrospective case series that compared outcomes with historical controls.^23^ The article by Stoot *et al* was a multicentre study comparing the ERP with both historical controls in the same centres before the introduction of the ERP or during the same period in other centres using traditional care.^20^ The two studies that described single interventions in one parameter but within an ERP compared outcomes in the study cohorts.^21,22^

The ERP protocol did vary between studies. However, all described a multimodal clinical pathway incorporating patient education, regional anaesthesia, optimal pain relief, judicious use of surgical drains (including nasogastric tubes and urinary catheters), early mobilisation and early introduction of oral liquids post-operatively (Tables 2 and 3).

**Table 2:** Summary of fast track multimodal elements in each study in pancreatic resectional surgery

	**Berberat *et al*^16^**	**Balzano *et al*^17^**	**Montiel Casado *et al*^23^**	**di Sebastiano *et al*^24^**
Pre-operatively		Information given to patient about fast track rehabilitation	Information given to patient; LMWH	Oral nutrition until 10pm; no premedication
Day 0	LMWH; octreotide; NG tube and drains used routinely; ICU stay; epidural or PCA	Thoracic epidural (T7-9; bupivacaine 0.125% and fentanyl 2µg/ml) plus IV paracetamol and NSAIDs	Epidural analgesia; removal of NG tube after surgery; ICU stay; liquids; prokinetic and octreotide	Analgesia by elastomeric pump*; remove NG tube on extubation; warm IV fluids; ICU stay; CVP <5mmHg
Day 1	Metoclopramide, lactulose and magnesium until first stool; oral fluids within 6h post-operatively	Remove NG tube if draining <300ml; mobilise out of bed; IV fluids until adequate oral intake	Move to ward; moving patient to chair; inhalation; liquid diet	Move to ward; mobilise four times daily; clear oral fluids within 4h post-operatively; metoclopramide and paracetamol
Day 2	Stepwise reduction in analgesia to non-opioids	Enhanced mobilisation (>2h out of bed)		Light diet; continue as per day 1
Day 3	Removal of drains between days 1 and 3; gradual increase in diet	Enhanced mobilisation (>4h out of bed); clear free fluids	Remove epidural; semiliquid diet; remove Foley catheter	Stop elastomeric pump; start NSAIDs; remove catheter; soft diet
Day 4		Solid food intake	Soft diet	Normal diet
Day 5	Diet increased daily until 1,000kcal on day 8; remove drain (if <200 ml); remove epidural	Discharge if no fever; good pain control and tolerance of oral analgesics	Discharged if no fever, pain control with oral analgesics, solid foods >1,000kcal/day; adequate mobilisation and willingness for discharge	Plan for discharge on day 7 if pain control with oral analgesics, no nausea, solid food; adequate mobilisation and willingness for discharge

LMWH = low molecular weight heparin; NG = nasogastric; ICU = intensive care unit; PCA = patient controlled analgesia; IV = intravenous; NSAID = non-steroidal anti-inflammatory drug; CVP = central venous pressure

* ketoprofen 960mg, tramadol 600mg, ranitidine 450mg, metoclopramide 90mg, morphine 15–30mg dissolved in 300ml saline solution

**Table 3:** Summary of fast track multimodal elements in each study in liver resectional surgery

	van Dam *et al*^18^	MacKay *et al*^19^	Stoot *et al*^20^	Koea *et al*^21^	Hendry *et al*^22^	Lin *et al*^25^
Pre-operatively	Oral nutrition until midnight; no premedication	Information given to patient about fast track rehabilitation	Information given to patient; no premedication; carbohydrate drink until 2h pre-operatively	Nil by mouth for 4h pre-operatively	Oral nutrition until midnight; no premedication	Information given to patient; no premedication or bowel preparation
Day 0	Thoracic epidural; remove NG post-operatively; no routine drains; oral fluids post-operatively; CVP <5mmHg	Oral fluids until 2h pre-operatively; no routine use of drains; oral fluids and supplementary drinks; PCA	Thoracic epidural catheter; no routine NG tube; oral liquid diet 6h post-operatively; laxatives and prokinetics; CVP <5mmHg	No routine use of NG tubes or surgical drains; liquid/light diet on waking	Thoracic epidural; remove NG post-operatively; no routine drains; free clear fluids post-operatively; out of bed for 2h	Thoracic epidural catheter; no routine drains or NG tube; oral liquid diet 6h post- operatively
Day 1	Mobilise; IV fluids stopped; normal diet; paracetamol and magnesium oxide	Diet If tolerated; small Gelofusine® boluses If hypovolaemic (stopped after 24h)	Mobilise; IV fluids stopped; normal diet; paracetamol and magnesium oxide	Remove arterial line and catheter; unrestricted diet; mobilise; routine blood tests	Mobilise; IV fluids stopped; normal diet; paracetamol	Mobilise >2h; reduce IV fluids; 1l liquid diet; catheter out
Day 2	As above	Remove PCA; step-down analgesia; remove catheter; mobilise	As above	Mobilise; continue diet; repeat blood tests	As above	Mobilise four times daily; epidural removed; NSAIDs
Day 3	Stop epidural; start NSAIDs; remove catheter; full oral intake	Mobilise; continue diet; repeat blood tests	Stop epidural; start NSAIDs; remove catheter; full oral intake	As above; first surgical dressing change	Stop epidural; start NSAIDs; remove catheter; full oral intake	Mobilise four times daily <6h; 21 light diet
Day 4	Review discharge criteria		Review discharge criteria	Review discharge criteria	Review discharge criteria	Oral medication; stop IV fluids; mobilise >6h
Day 5				Check blood tests; remove central venous line; discharge	
Normal diet; give discharge instructions; mobilise four times daily >6h			Discharged If pain control with oral analgesics and solid foods; adequate mobilisation	Discharge when normal or decreasing bilirubin, good pain control, normal diet tolerated and mobilising to pre-operative level		Discharge on day 6 when fully mobile, pain control adequate and normal organ function; follow-up In outpatients clinic on days 10, 15 and 30

NG = nasogastric; CVP = central venous pressure; IV = Intravenous; NSAID = non-steroidal anti-inflammatory drug; PCA = patient controlled analgesia

The demographics and study outcomes of individual articles are shown in Tables 1,4 and 5. The two studies that described single interventions in one parameter but within an ERP have an overall value described that includes all investigational cohorts of patients with the ERP. Tables 4 (pancreatic resections) and 5 (liver resections) demonstrate consistently reduced length of post-operative stay in both liver and pancreatic resectional surgery with the incorporation of an ERP. This reduced length of stay is in comparison to both the studies’ controls and historical controls.^10–14^ In the studies involving liver resections, two articles specified intra-operative blood loss ranging between a mean of 50ml and 760ml.^20,25^ This was significantly less than with traditional care in one study.^20^ In the pancreatic resection studies, intra-operative blood loss ranged between 300ml and 700ml and was specified in 3 out of 4 articles.^17,24^ In the study by Balzano *et al*, this blood loss was not significantly reduced when compared with a historical control.^17^

**Table 4:** Outcomes of studies implementing fast track pancreatic resectional surgery

Authors	NG tube removed	Feeding	Gastrointestinal function	Abdominal drains	Urinary catheter removed	Length of post-operative stay	Morbidity	Mortality	Readmission rate
Berberat *et al*^17^	80.4% removed post-operatively; 13.3% removed on day 1; reinsertion rate 11.4%	First liquid 1 day (0–6 days); complete oralisation 5 days (1–24 days)	First stool 4 days (1–9 days)	3 days (0–19 days)	5 days (1–49 days)	10 days (4–115 days)	41.2%	2%	3.5%
Balzano *et al^17^*	92.9% removed on day 1; 84.2% did not require reinser-tion	All patients without NG tube In situ commenced liquid diet on day 3 and food on day 4	First flatus 3 days (1–6 days); first stool 5 days (1–9 days)	Not stated; percutaneous drainage required In 3.6%	Not stated	13 days (7–110 days)	47.2%	3.6%	7.1%
Montiel Casado *et al*^23^	Removed after surgery; not stated if needed reinser-tion; delayed gastric emptying in 2.4%	Actual outcomes not stated	Not stated	Not stated	Not stated	11 days (4–18 days)	47.6%	4.9%	14.6%
di Sebastiano *et al*^24^	Removed within a few hours of surgery in 24.1%; on day 1 in 42.1%; later in 33.8%	First liquid 1 day (0–8 days); complete oralisation 5 days (3–11 days)	First flatus 3 days (1–7 days); first stool 5 days (2–9 days)	5 days (3–23 days) for right drain and 6 days (3–29 days) for left drain; 7 patients discharged with drain	3 days (1–9 days)	10 days (6–69 days)	38.6%	2.7%	30-day rate: 6.2%

NG = nasogastric

**Table 5:** Outcomes of studies implementing fast track liver resectional surgery

	NG tube removed	Feeding	Gastrointestinal function	Abdominal drains	Urinary catheter removed	Length of post-operative stay	Morbidity	Mortality	Readmission rate
van Dam *et al*^18^	NG tube inserted at induction in 78.7%; removed within 4h of surgery; reinserted in 3.28%	92% had oral intake within 4h post- operatively; normal diet In 1 day (0–3 days)	Not stated; 5% constipated after day 3	2% had Intra-operative drains; not stated when removed	Not stated	6 days (3–82 days)	41%	0%	13%
MacKay *et al*^19^	Not stated	Not stated	First stool 4.5 days (data only available In 83.3% of patients); flatus not stated	Not used in any patient	Not stated	4 days (2–7 days)	25%	0%	Not stated
Stoot *et al*^20^	No NG tubes inserted	Normal diet in 1 day (1–2 days)	Not stated	No drains Intra-operatively	Not stated	5 days (3–10 days)	1%	0%	0%
Koea *et al*^21^	Not stated	Regular diet on day 1 in 26%	First flatus passed on day 1 in 25%	2% had drains Intra-operatively; not stated when removed	Not stated	4.6–7.2 days	16–22%	0%	3%
Hendry *et al*^22^	Aimed to remove all post-operatively; not stated number reinserted	First liquid on day 0 in 94%; diet on day 1 in 37% and day 2 in 91%	First flatus 3 days (2–4 days); first stool 5 days (4–6 days)	13% had intra-operative drains	Not stated	6 days (4–7 days)	30-day rate: 25%	30-day rate: 3%	7%
Lin *et al*^25^	Not Inserted pre-operatively; inserted for complications in 3.57%	Not stated	Not stated	Not stated	Not stated; one patient required intra-abdominal drain for bile leak	7 days (3–26 days)	46.4%	1.8%	7.1%

NG = nasogastric

Six studies described the return of gut function after surgery within an ERP.^16,17,19,21,22,24^ In the liver resection group, the first flatus passage was at days 3–5 and the first stool at days 4–5.^16,17,19^ One article compared first stool with a historical control and found a more rapid return of gut function of 1 day (*p*<0.001).^17^ In the pancreatic resection group, stool was passed at days 4.5–5^19,22^ and flatus on day 3 post-operatively.^22^ In the article by Koea *et al*, which investigated different analgesics within an ERP, all patients receiving intrathecal morphine passed flatus on post-operative day 1 (*n*=50).^21^ In the epidural group, 12 passed flatus on day 1, 28 on day 2 and 10 on day 3 (*n*=50). Stool passage was not documented.

## Discussion

This article aimed to review the current evidence for implementing an ERP in HPB surgery. It demonstrates that the incorporation of such protocols appears feasible and safe. Most notably, the length of post-operative stay can be reduced significantly. However, whether this is at the expense of increased rates of readmission is unknown at present due to the limited number of trials. While the ERP has been the topic of numerous trials in colorectal surgery, scanty reports exist for its efficacy in HPB surgery.

Many of the principles of the ERP have been extracted from ERPs in colorectal surgery. As a result, it is possible that these principles cannot be transcribed so easily to HPB surgery. Procedures may be more complicated and may involve longer lengths of post-operative stay because of this. Differences exist, for example, in pre-operative fluids. In liver surgery a relative hypovolaemia, low central venous pressure and avoidance of excessive pre-operative fluids is preferred to minimise intra-operative blood loss.

Minimally invasive surgery is often included as part of an ERP in colorectal surgery although its positive effects are yet to be proved conclusively.^5^ Laparoscopic liver resection is under investigation and currently the topic of many reviews.^20,26^ Hospital stays of five days have been reported following major resections for benign disease.^27^ While minimally invasive surgery does reduce morbidity secondary to large upper abdominal incisions, the application of regional anaesthetic techniques and optimum analgesic control in open surgery can also reduce hospital stay. Indeed, in colorectal surgery, laparoscopic resection is being challenged by open surgery in the setting of an ERP,^28^ with one RCT demonstrating no difference in mortality, morbidity, readmission rate or hospital stay.^29^

Concerns over the safety of laparoscopic HPB surgery remain due to reported rates of conversion of 8–15% secondary to haemorrhage and margin positive rates of 2%.^30^ In addition, there are the concerns of pneumoperitoneum increasing the risks of tumour dissemination and the additional incisions needed to remove large specimens.^31^

While laparoscopic liver resection is now used widely in most HPB centres, especially for atypical or wedge resections, the adoption of laparoscopic surgery for major pancreatic resections has not advanced at an equivalent rate. In particular, the application of laparoscopic surgery for complex procedures such as pancreaticoduodenectomy, even in leading institutions for robotic surgery, has not demonstrated an improvement in length of stay or morbidity, which would justify the widescale adoption of these techniques.^32^

Another contentious issue to many pancreatic surgeons will be the ERP’s minimal use of intra-operative abdominal drains. Many see correctly positioned drains as essential in recognising life threatening post-operative complications such as anastomotic breakdown and haemorrhage. Perhaps this principle of ‘no abdominal drain’ use, transcribed from the ERP in colorectal surgery, cannot be applied so easily in HPB surgery. While it is not the purpose of this review to appraise evidence for individual parameters of the ERP, this again serves to highlight the differences from colorectal fast track surgery. Perhaps of greater importance to the pancreatic surgeon is a protocol for early versus late drain removal or even no drain placement for patients deemed lower risk for an anastomotic leak. This issue has been the subject of several publications.^33–35^

A further contentious issue is that of post-operative feeding. Concerns in particular surround protecting the pancreatic anastomosis following pancreatic resection. The articles in this review implementing early oralisation (some combined with octreotide) as part of an ERP have not shown any increase in complication rate.^16,17,24,23^ Many surgeons nevertheless remain committed to a post-operative period of ‘bowel rest’, with the theory that it will reduce the risk of anastomotic leak.

Of concern in studies evaluating the efficacy of implementing the ERP is the choice of primary outcome. Frequently, studies used length of hospital stay. This may not, however, best reflect the quality of functional recovery. The Cochrane review of the ERP in colorectal surgery concluded that there was no proof that the use of this endpoint was a medically important parameter and that complication rates may be a better quantitative measure of safety.^5^ We therefore propose that the implementation of a standardised multimodal protocol in HPB surgery that increases awareness of goals that improve safety and clinical outcomes is of greater importance.

As evidenced by the Cochrane meta-analysis, simply implementing an ERP does not ensure improved results.^5^ What is more important is that there is stringent overseeing of protocol adherence by all members of the multidisciplinary team together with continued alertness for decreasing compliance. Implementing and auditing such protocols tailored for the HPB surgeon has been demonstrated to be safe. Emphasis must surely now be placed on any attempt to reduce morbidity from such high risk intervention by the introduction of standardised care protocols.

## Conclusions

The introduction of an ERP in HPB surgery appears safe and feasible. Currently, many of the principles of the multimodal pathway are derived from the colorectal ERP and distinct differences exist that may inhibit its uptake among HPB surgeons. RCTs are needed to clearly define evidence-based parameters in this complex group of patients.
